# Acupuncture for prostatectomy incontinence: study protocol for a multicenter single-blind randomized parallel controlled trial

**DOI:** 10.1186/s13063-021-05805-5

**Published:** 2022-01-04

**Authors:** Yao Zhang, Shanqi Guo, Chaoran Wang, Xiaodi Liu, Yan Liu, Hongcai Shang, Peiying Yang, Liang Wang, Jingbo Zhai, Xiaojiang Li, Yingjie Jia

**Affiliations:** 1 First Teaching Hospital of Tianjin University of Traditional Chinese Medicine, Tianjin, China; 2 Tianjin University of Traditional Chinese Medicine, Tianjin, China; 3 National Clinical Research Center for Chinese Medicine Acupuncture and Moxibustion, Tianjin, China; 4 Key Laboratory of Chinese Internal Medicine of Ministry of Education, Dongzhimen Hospital, Beijing University of Chinese Medicine, Beijing, China; 5 Tianjin Medical University General Hospital, Tianjin, China

**Keywords:** Acupuncture, Post-prostatectomy incontinence, Efficacy, Randomized control trial, Study protocol

## Abstract

**Background:**

Urinary incontinence is a common complication post radical prostatectomy. Acupuncture is considered an effective treatment for post-prostatectomy incontinence (PPI), but the evidence is still limited. We propose to evaluate the effectiveness of acupuncture in a rigorously conducted trial.

**Methods:**

Twenty hospitals will recruit 340 participants with urinary incontinence after radical prostatectomy in China from April 2021 to April 2022. Participants will be randomly allocated to acupuncture or sham acupuncture with a 1:1 ratio using computerized simple random sampling. The study plan consists of 1-week baseline, 6-week treatment, and 18-week follow-up. Eighteen 30-min sessions of acupuncture or sham acupuncture treatment will be provided between weeks 1 and 6. The primary outcome is the change in the International Consultation on Incontinence Questionnaire-Urinary Incontinence Short Form (ICIQ-UI-SF) score at the week 6 from the baseline. Secondary outcomes include the change in volume of urine leakage at weeks 4 and 6 from a baseline measured using the 1-h pad test; 72-h incontinence episode frequency based on a 72-h voiding diary; change in the Expanded prostate cancer Index Composite scale (EPIC-26); change in the Self-Rating Anxiety Scale; weekly consumption of pads; and the severity of urinary incontinence based on a 72-h bladder diary and self-assessment of the therapeutic effect. The safety of acupuncture will also be assessed.

**Discussion:**

This trial will help to identify whether acupuncture is effective for PPI, and, if so, whether it exerts a therapeutic rather than a placebo effect.

**Trial Registration:**

www.Chictr.org.cnChiCTR2100042500. Retrospectively registered on 22 January 2021.

**Supplementary Information:**

The online version contains supplementary material available at 10.1186/s13063-021-05805-5.

## Background

Prostate cancer is one of the most common malignant tumors of the male genitourinary system and radical prostatectomy is the first-line treatment for localized prostate cancer [1]. Post-prostatectomy incontinence (PPI) is a known complication with an incidence rate ranging from 4% to 31% [2], which has a significant impact on men’s quality of life (QoL) [3, 4]. The complication may generate feelings of low self-esteem, anxiety, and depression. The patients’ shame and discomfort arise from the inability to control the bladder. Patients who do not wish to wear a diaper daily, but always carry it for safety in their bag when leaving home [5]. The present study revealed that the postoperative period of 2 to 6 months had a severe impact on their QoL [6].

The treatments for PPI are mainly divided into two types. Conservative treatment includes bladder or pelvic floor muscle training [7, 8], biofeedback [9, 10], and drug therapy [11, 12]. Surgical intervention mainly includes artificial urethral sphincter implantation [13], urethral suspension [14, 15], or injection therapy [16]. All these treatments may benefit patients; Although, it cannot be denied that the selective serotonin reuptake inhibitor drugs such as duloxetine, which is often accompanied by symptoms of insomnia, nausea, loss of appetite, irritability, and other side effects [11, 17]. Further, surgical interventions are traumatic forms of treatment and are also costly [18]. Pelvic floor muscle training is the most used conservative treatment for urinary incontinence, but for elderly male patients with prostate cancer, whether functional training is carried out correctly is difficult to evaluate and assess, and it is challenging to implement.

Acupuncture is a typical treatment modality of traditional Chinese medicine (TCM) [19, 20]. With few adverse effects, TCM may be used as an alternative treatment approach for patients. Many research centers, mostly in China, have conducted clinical trials to evaluate the safety and effectiveness of acupuncture in managing PPI. Zhishun Liu and Yan Liu et al. [21] conducted a multicenter, randomized clinical trial and revealed that women with stress urinary incontinence can benefit from electroacupuncture involving the lumbosacral region, compared with sham electroacupuncture, resulting in less urine leakage after 6 weeks; the effects persisted 24 weeks after treatment and the incidence of adverse events was low. Huan Chen and Yu Liu et al. [22] in a meta-analysis of seven studies including 830 men with PPI showed that acupuncture can significantly improve the score of the International Consultation on Incontinence Questionnaire-Urinary Incontinence Short Form for men with urgent urinary incontinence (UUI) when compared with medicine, and no adverse event was reported in the studies. Therefore, acupuncture could be considered as an effective treatment for urinary incontinence.

However, the quality of evidence has been low and inconclusive. Therefore, we plan to perform a multicenter randomized controlled trial (RCT) to investigate the efficacy and safety of acupuncture in the treatment of PPI in patients with prostate cancer.

## Methods

### Study design

This study is a multicenter, single-blind, randomized parallel controlled prospective clinical trial with the objective of estimating the efficacy and safety of acupuncture on PPI by comparing a verum acupuncture group with a sham acupuncture group. Participants considered suitable for the study will be randomly allocated into the intervention group (verum acupuncture) or the control group (sham acupuncture) in a 1:1 ratio. This is a clinical trial of superiority.

### Study participants

#### Population

A target sample of 340 participants will be recruited from 20 hospitals across China: Tianjin Medical University General Hospital; The Second Hospital of Tianjin Medical University; Tianjin Medical University Cancer Hospital; Tianjin First Central Hospital; Tianjin Third Central Hospital; Peking University Third Hospital; Dongzhimen Hospital affiliated to Beijing University of Chinese Medicine; West China Hospital of Sichuan University; Benxi Central Hospital; First Affiliated Hospital of Dalian Medical University; The Second Hospital of Dalian Medical University; The First Affiliated Hospital of Kunming Medical University; Qinghai University Affiliated Hospital; Jiangsu Province People’s Hospital; First Hospital of Shanxi Medical University; Shandong Province Hospital; The Fifth People’s Hospital of Jinan; Inner Mongolia People’s Hospital; The Second Hospital of Anhui Medical University; and Guizhou Province People’s Hospital. Informed consent will be obtained from all participants before randomization.

#### Baseline assessment

A baseline registration will be performed before treatment. Participants will provide a review of basic information, malignancy history including operation history, pathology, and QoL assessment including urinary incontinence. A safety evaluation will also be performed.

#### Recruitment

We will recruit participants who are attending the 20 abovementioned hospitals in this trial. We will use WeChat to publish recruitment information, and use the “recruiters” applet to recruit and retrieve information for patients. Before recruiting patients, the researchers of each sub-center will explain the research protocol to potential trial participants and ask the patients sign the informed consent. The informed consent is in duplicate, one is owned by the participant or authorised surrogate and the other one is owned by the researcher. Finally, all the informed consent forms of each sub-center will be submitted to the project leader.

#### Inclusion criteria

Patients who meet all the following requirements will be allowed to enroll:

1. Patients who have received radical prostatectomy with a definite pathological diagnosis of prostate cancer:
2. Patients with urinary incontinence symptoms, which refer to the meaning of urinary incontinence by the International Continuity Society or evaluated by the urologists as experiencing urinary incontinence resulting from radical prostatectomy [23];
3. Karnofsky Score ≥ 60 or Eastern Cooperative Oncology Group (ECOG) Score 0–2; and
4. Willing to participate in the study and sign the consent forms.

#### Exclusion criteria

Patients who meet all the following criteria will be excluded:

1. Urgency urinary incontinence caused by detrusor overactivity and bladder spasm, and urinary incontinence caused by neurogenic urethral dysfunction;
2. Urinary incontinence treated by cystostomy, urethral sphincter reconstruction, or urethral suspension;
3. Currently receiving less than 6 months treatment similar to acupuncture (such as electroacupuncture, warm moxibustion, and warm acupuncture);
4. Judged by urologists as complete urinary incontinence;
5. Urinary tract infection (except asymptomatic bacteriuria);
6. History of severe arrhythmia, severe cardiac insufficiency, acute myocarditis, constrictive pericarditis, pericardial tamponade, severe valvular disease, heart failure, or other serious heart diseases;
7. History of severe liver injury or potentially severe liver disease (ALT or AST > 10 times normal);
8. History of severe renal impairment (estimated GFR < 25 mL/min/1.73 m^2^);
9. Known to coagulation dysfunction (with typical clinical diagnosis or clear laboratory test results);
10. Patients with mental illness or cognitive impairment, or severe depression;
11. Patients who are not suitable to participate in this study or who are likely to withdraw from the study based on the judgment of the researchers; and
12. Patients participating in other clinical trials.

#### Withdrawal from the study

Participants will be allowed or asked to withdraw from the study in the following circumstances:

1. Patients who express an unwillingness to being subjected to the assigned treatment for various reasons at any stage of the trial;
2. Patients experience an adverse event (AE) that necessitates their withdrawal from the trial;
3. Patients who cannot fully participate in the treatment or follow-up period; and
4. Patients can withdraw from the study voluntarily for any reason.

#### Randomization and blinding

A total of 340 participants will be randomly allocated into the treatment group or the control group. Each hospital will recruit 17 patients. In this trial, we will carry out block randomization in a 1:1 ratio according to the sequence generated using SAS software version 9.4 (SAS Institute Inc. Cary, NC). Random allocation will be performed after eligible participants consent in written form and complete baseline assessments. The central randomization system provided by the third party was used to generate the allocation sequence (http://zysj.clindacro.com/login.aspx), and the sub-center researchers (urologists) logged in the randomization system after screening the patients, input the account number and password, input the basic information of the subject according to the prompt, and the random system will give the subject code and random numbers. The sub-center therapists (acupuncturists) log into the central randomization system through their own account and password to check the subject codes, the random number, and treatment groups (acupuncture/non-meridian and non-acupoint shallow acupuncture). The participants, outcome assessors, and statisticians will be blinded to treatment allocation. The acupuncturist will not be blinded to the participant’s allocation. All participants in the study will sign a confidentiality commitment to avoid blind disclosure.

#### Ethics

The study will be performed in accordance with the principles of the Declaration of Helsinki and has been approved by the ethics committee boards of the participating hospitals. Written informed consent will be obtained from each subject before the patients are enrolled in the trial.

### Interventions

**Fig. 1:**
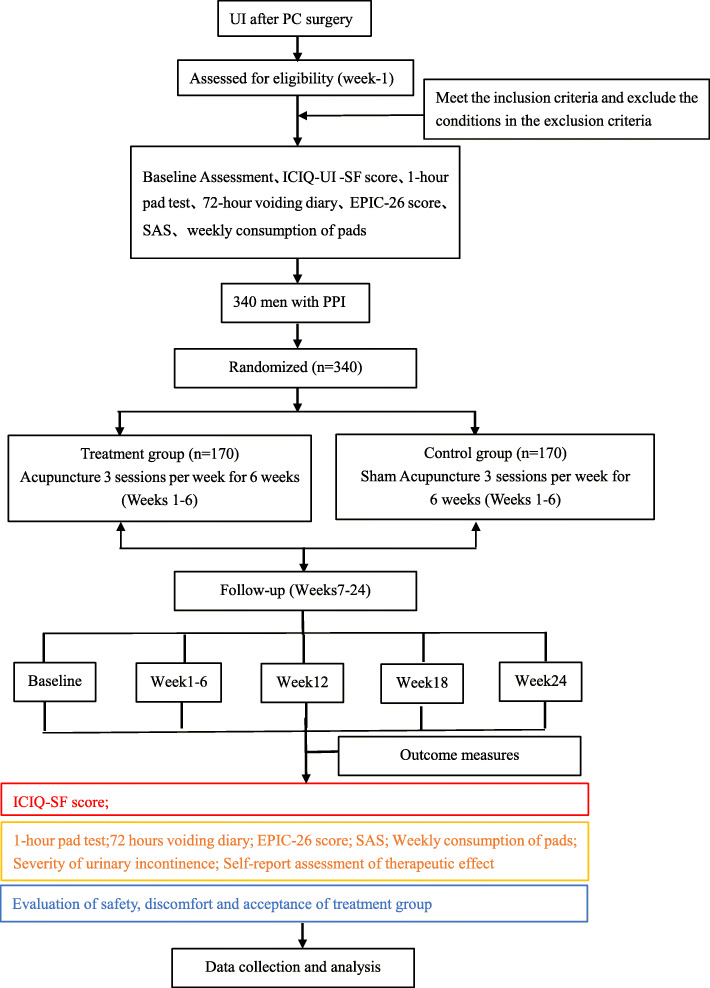
Trial flowchart. UI, urinary incontinence; PC, prostate cancer; PPI, prostatectomy incontinence

According to the predetermined randomization results, participants will be randomly allocated to the verum or sham acupuncture group. All participants will go through a standardized interview, which will be recorded on the case report forms. To minimize bias, all the acupuncturists in this trial are specialists in acupuncture. They have a minimum of 2 years experience in acupuncture treatment and hold licenses to perform acupuncture. Before performing this trial, all acupuncturists will receive special training regarding the purpose and content of the trial, treatment strategies, and quality control. The flowchart and study design schedule are presented in Fig. 1 and Table 1, respectively.

**Table 1:** Study design schedule

Contents	Research period								
	Screening	Baseline	Treatment (W1–6)				Follow-up (W7–24)		
	W−1	W0	W1	W2	W4	W6	W12	W18	W24
**Visit**	V0	V1	V2	V3	V4	V5	V6	V7	V8
**Name**	×	×							
**Age**	×	×							
**Contact information**	×								
**Inclusion/exclusion criteria**	×								
**Informed consent**	×								
**Screening dispose**	×								
**Randomization**	×								
**Vital signs**^**1**^	×	×	×	×	×	×			
**Blood routine**^**2**^	×	×				×			
**Urine routine**^**3**^	×	×				×			
**Biochemical items**^**4**^	×	×				×			
**ECG**^**5**^	×	×				×			
**Height**		×							
**Weight**		×							
**BMI**^**6**^		×							
**Education and occupation**		×							
**Karnofsky score**		×							
**ECOG score**^**7**^		×							
**Personal history**^**8**^		×							
**Past histories**		×							
**Physical examination**^**9**^		×	×	×	×	×			
**Diagnosis time**		×							
**Pathological type**		×							
**Gleason score**		×							
**Clinical stages**		×							
**Operation type**		×							
**Treatment of PPI**		×	×	×	×	×	×	×	×
**Treatment of PCa**		×	×	×	×	×	×	×	×
**Daily water consumption**		×	×	×	×	×	×	×	×
**Drink preference**		×	×	×	×	×	×	×	×
**Postoperative extubation time**		×							
**Time of incontinence**		×							
**ICIQ-UI SF**^**10**^		×				×	×	×	×
**1-h pad test**		×			×	×			
**72-h voiding diary**		×	×	×	×	×	×	×	×
**EPIC-26**^**11**^		×				×	×	×	×
**SAS**^**12**^		×				×	×	×	×
**Weekly consumption of pads**		×	×	×	×	×	×	×	×
**Severity of PPI**		×				×	×	×	×
**Self-assessment**						×	×	×	×
**Blind assessment**						×			
**Major AE of PCa**^**13**^			×	×	×	×	×	×	×
**AE**^**14**^			×	×	×	×	×	×	×
**SAE**^**15**^			×	×	×	×	×	×	×

Notes:

^1^Vital signs: blood pressure, pulse, body temperature, and respiration

^2^Blood routine: hemoglobin, red blood cell count, white blood cell count, lymphocyte, neutrophil, and platelet count

^3^Urine routine: white blood cell, red blood cell, protein, and ketone body

^4^Biochemical items: alkaline phosphatase, alanine aminotransferase (ALT), aspartate aminotransferase (AST), total bilirubin, urea nitrogen, glomerular filtration rate, creatinine, glucose, triglyceride, total cholesterol, high-density lipoprotein, low-density lipoprotein, potassium, sodium, calcium

^5^ECG: electrocardiogram

^6^BMI: body mass index

^7^ECOG score: Eastern Cooperative Oncology Group score

^8^Personal history: including family history, smoking history, drinking history, drug and food allergy history, marriage history, occupational exposure history

^9^Physical examination: the researchers will conduct a comprehensive physical examination at baseline, including general appearance, skin, neck (including thyroid), eyes, ears, nose, throat, lung, heart, abdomen, back, lymph nodes, limbs, and nervous system examination. All other visits will have a brief physical examination, i.e., a general appearance examination

^10^ICIQ-UI SF: International Consultation Incontinence Questionnaire-Urinary Incontinence Short Form

^11^EPIC-26: The Expanded Prostate Cancer Index Composite 26-item version

^12^SAS: Self-Rating Anxiety Scale is a 20-item self-report assessment

^13^Major adverse event (AE) of prostate cancer (PCa): it refers to the AEs related to the progression of PCa from the time of enrollment to the end of follow-up

^14^AE: It refers to the AEs caused by the intervention measures of the project from the time of admission to the end of follow-up

^15^SAE: It refers to the serious adverse events caused by the intervention measures of the project during the period from enrollment to the end of follow-up

The trial will last 24 weeks, including treatment and follow-up periods. All participants will receive treatment for 6 weeks. Participants in both groups will receive verum or sham acupuncture three times a week (recommended interval of one day). Each acupuncture session requires approximately 20 min to perform. Sterile needles for single use (0.25 × 40 mm; Jiangsu Medical Supplies Factory Co., Ltd.) will be used in this study. Needles in the treatment group will be inserted at a depth of 0.8-1 inches and manually manipulated by rotation methods. After acupuncture, the patient's "De Qi" should be taken as the criterion (the feeling of pain and numbness). In the control group, needles will be inserted about 0.5 inch in depth and will not receive further manipulation. To prevent blind uncovering, the operator will pay attention to the depth and angle of the shallow stab. All participants will be evaluated by blind method at week 6 after acupuncture. The Blind Method Assessment is shown in Additional file 1.

All participants need to stay in the hospital for observation for at least 10 min after acupuncture, and can leave only with no discomfort.

In order to ensure the safety of participants, in case of emergency events, it is necessary to break the blindness in advance if there is any adverse event which could not be judged whether it is related to acupuncture intervention. When the blinding is broken, the time, reason, and personnel performing the blind breaking ahead of time shall be recorded and the supervisor shall be notified in time. Once the blinding is broken, the patient would not continue to participate in the study, and the tested data should not be adopted in this study. However, the safety analysis data should still be included.

### Acupoints used in the treatment group

The stimulation points in the intervention group will be RN12 (Zhongwan), RN6 (Qihai), ST36 (Zusanli, bilaterally), SP6 (Sanyinjiao, bilaterally), SP9 (Yinlingquan, bilaterally), SP10 (Shuidao), RN3 (Zhongji), and RN2 (Qugu). All acupoints are located according to the National Standard of People's Republic of China (GB/T 12346-2006). The acupoint diagram is shown in Additional file 2.

### Sham acupoints used in the control group

To ensure the quantity of stimulus is uniform between two groups, the same kind, size, and the number of needles will be used for the control group. However, the control group will be treated with shallow needling at the non-meridian and non-acupoint positions. The acupuncture points shifted 1-inch from the actual location and have no therapeutic value.

### Permitted and prohibited concomitant treatments

Both groups can receive conventional treatment for prostate cancer (such as endocrine therapy) and/or pelvic floor muscle training, which differ from acupuncture principles. Researchers need to keep a detailed record. Treatments like the principles of acupuncture are not allowed.

### Outcome measures

#### Primary outcome measures

##### International Consultation Incontinence Questionnaire-Urinary Incontinence Short Form (ICIQ-UI-SF)

Scores of the scale will be calculated to investigate the incidence of urinary incontinence and the impact of urinary incontinence on patients. It will be evaluated at baseline, and at 6, 12, 18, and 24 weeks post treatment. The scale is shown in Additional file 3.

#### Secondary outcome measures

##### One-hour pad test

It is a test to objectively evaluate urinary incontinence. It is measured to assess the change in volume of urine leakage from baseline at weeks 4 and 6.

##### Seven-two-hour voiding diary

On the basis of not changing life status and urination habits, 72 h of fluid intake and urination time are continuously recorded every week. It is used to assess the change in mean 72-h incontinence-episode frequency (IEF) from baseline and at weeks 1, 2, 4, 6, 12, 18, and 24. The scale is shown in Additional file 4.

##### EPIC-26

The Expanded Prostate Cancer Index Composite 26-item version includes three categories of questions aimed to evaluate the prostate function and QoL of patients with prostate cancer in the past 4 weeks. Evaluated at 6, 12, 18, and 24 weeks, changes in scores from the baseline will be analyzed. The scale is shown in Additional file 5.

##### SAS

Self-Rating Anxiety Scale is a 20-item self-report assessment used to measure anxiety levels, based on the scoring of 4 groups of symptoms: cognitive, autonomic, motor, and central nervous system symptoms. The scores will be evaluated at 6, 12, 18, and 24 weeks, and changes in scores from the baseline values will be analyzed. The scale is shown in Additional file 6.

##### Weekly consumption of pads

Changing and average weekly consumption of pads at 1, 2, 4, 6, 12, 18, and 24 weeks from the baseline will be calculated.

##### Severity of urinary incontinence

It will be evaluated at 6, 12, 18, and 24 weeks. The change in the number and percentage of severity ratings from the baseline value will be analyzed.

##### Self-report assessment of therapeutic effect

A 4-point scale is used: no help, little help, medium help, and great help [24]. Treatment effects will be evaluated at 6, 12, 18, and 24 weeks.

### Safety assessment

To exclude any related serious diseases, patients will be asked to undergo necessary inspection before randomization, including vital signs, blood routine, urine routine, biochemical items, ECG. These tests will be performed at the week 6 of the study to evaluate the safety of this trial. If any AEs occur during the trial, patients will be treated as soon as possible.

### Adverse events

With any adverse events during treatment, protective therapeutic measures shall be taken in time to ensure the safety of participants. According to the different levels of adverse events, researchers will make the judgment if the participants will continue, suspend, or withdrawal from the study. Common treatment-related AEs including fainting during acupuncture, local bleeding, sticking of needle, subcutaneous hematoma, and itching at the sites of needle insertion. If the patient has an adverse event due to acupuncture and the investigator decided that the patient would not suitable to continue to participate the trial, the patient will be weeded out consequently.

### Data management

All information related to this study, including but not limited to the following documents: research protocol, informed consent, CRF, investigator manual, training materials, research protocol, subject grouping information, statistical report, and research conclusion, must be strictly confidential. All personnel involved in the study shall sign a confidentiality undertaking. The CRF is composed of paper form and electronic form. The monitor (CRA) shall check out the CRF on site to ensure the consistency between the paper CRF and the electronic CRF regularly. The revealed problems shall be corrected and the name of the corresponding researcher should be signed after verification with the researcher. In order to ensure the accuracy of data, the researcher shall carefully fill in the CRF paper and upload the data into the electronic CRF. The revealed problems during the uploading process should be registered and reported to CRA. After all signed documents match the requirements of the research protocol, the database will be locked by the data management unit, and no data can be modified after locking the database.

### Quality control

The design and implementation of multicenter clinical research require the cooperation of departments, and we established committees as listed below: ① The Expert Committee: the expert committee participated by clinical experts, statistical experts, and quality control experts. They are responsible for building the principles of clinical research methodology and ensure its scientificity, rationality, and feasibility of the protocol; they solve the major problems in the enforcement process of clinical research. ② The Executive Committee: the members have practical experience in clinical research, familiar with clinical research operation procedures and specifications, and they also possess the ability of medical information retrieval, communication, coordination, and organization. They are responsible for formulating of a research plan and informed consent and complete the project initiation application procedure. ③ The Data and Safety Monitoring Committee (DSMC): which is composed of independent third-party experts and does not belong to any institution of this study, to independently evaluate safety indicators and put forward audit suggestions based on the analysis results of safety indicators, thereby protecting the rights and security of participants. During the implementation of this study, members of the committee will regularly receive the research progress report issued by the Executive Committee every 3 months and hold open meetings to listen to the research process report from the research project leader. ④ The Data Management and Statistical analysis Center: its responsibilities include the establishment of data randomization systems and electronic database, case report form (CRF) design, data analysis, and verification.

### Statistical methods

#### Sample size calculation

By enrolling approximately 340 (170 in each group) participants, the study will provide 90% power to detect a between-group difference of 2.5 in reduction of ICI-Q-SF score from baseline using a 2-sided alpha level of 0.05, and assuming a common standard deviation of 6.3 and a dropout rate of 20%. A difference of 2.5 points in the ICI-Q-SF score was selected for sample size calculations, which is indicative of a clinically significant change [24–26].

#### Statistical analysis

Summary tables (descriptive statistics and/or frequency tables) will be provided for all variables as appropriate. The primary outcome analysis will use the Cochran-Mantel-Haenszel (CMH) test. For continuous variables, means and standard deviations will be presented, unless the variable has a skewed distribution, in which medians, 25th and 75th percentiles will be presented. For other categorical data, between-group comparisons will be performed using a Wilcoxon rank-sum test, chi-square test, or Fisher’s exact test, as appropriate. AE incidences for each treatment group will be tested by the *χ*^2^ test or Fisher’s exact test as appropriate. All the statistical analysis will be performed using SAS version 9.4 (SAS Institute Inc) with 2-sided tests at a significance level of < 0.05. We will use the multiple imputation to handle missing data.

## Discussion

PPI has a significant impact on the QoL of patients who underwent radical prostatectomy. It is closely associated with the trauma caused by surgery [27, 28]. Although urinary incontinence after prostate cancer heals within half a year, the need for active improvement of symptoms and the QoL of patients is widely recognized [29]. Surgical resection of prostate cancer mainly includes bladder neck, bilateral vas deferens ampulla, bilateral seminal vesicles, and the intact prostate. Although a complete resection of the tumor and avoidance of positive surgical edge can be achieved, the bladder neck and part of the prostatic apex urethra are always removed, which will lead to the urinary control structure damage. In addition, due to destructive surgical operate, intraoperative bleeding, tumor infiltration, and tissue adhesion, the distal sphincter, fascia, ligament, and pelvic floor muscles may be damaged, which leads to postoperative urinary incontinence [30, 31]. The efficacy of acupuncture has been proved by many RCTs [32]. A systematic review demonstrated that acupuncture was an appropriate adjunctive therapy for PPI, but the supporting evidence was insufficient. These limitations included small sample size, greater-than-anticipated withdrawal, the lack of a control group, and unclear statements about randomized allocation. Therefore, much stronger evidence is still needed to verify the efficacy and safety of acupuncture for PPI. We have presented the design of this RCT mainly focuses on the therapeutic effects of verum acupuncture compared with sham acupuncture on PPI. Completion of this trial will contribute to verifying the efficacy of acupuncture for the treatment of PPI. This multicenter study covers 13 provinces and cities from south to north of China. We hope that the results will provide robust evidence to support the application of acupuncture in the treatment of postoperative incontinence of prostate cancer.

## Trial status

The trial was registered on www.Chictr.org.cn. ChiCTR2100042500. This trial is currently recruiting participants and will be completed by 31 December 2022.

Protocol completed date: 10 May 2021

Protocol amendment number: 03

## Supplementary Information

**Additional file 1.** The Blind Method Assessment

**Additional file 2.** The acupoint diagram

**Additional file 3.** The ICIQ-UI-SF Scale

**Additional file 4.** 72-hour Voiding Diary

**Additional file 5.** The EPIC-26 scale

**Additional file 6.** The Self-Rating Anxiety scale
