# Supplementary material for: Acupuncture for prostatectomy incontinence: study protocol for a multicenter single-blind randomized parallel controlled trial
Source: Trials. 2022 Jan 4;23:9. doi: 10.1186/s13063-021-05805-5 (PMC8725553; doi:10.1186/s13063-021-05805-5)
Supplement: Supplementary file 1 — Additional file 1. The Blind Method Assessment [file 13063_2021_5805_MOESM1_ESM.pdf]

Blind Method Assessment

Name:____________ Random number:___________ Date:_________

Please answer the following questions according to your latest acupuncture experience.

1.How's your feeling about needle sensation (sensations induced by acupuncture, like sourness, numbness, distending, heaviness, etc)

Please ring a number between 0 (no needle sensation) and 10 (unbearable needle sensation).

0	1	2	3	4	5	6	7	8	9	10

None ←——————————————→ Unbearable

2.Do you have acupuncture experience? (Yes No)

3.Do you think you have needled at acupuncture points? (Yes No)

4.Are you sure you receiving acupuncture treatment? (Yes No)

The First Teaching Hospital of Tianjin University of Traditional Chinese Medicine for study design and all participating units for collection and management, the third party for analysis, interpretation of data; China Academy of Chinese Medical Sciences and China Center for Evidence Based Traditional Chinese Medicine as the guidance units.
