# Supplementary figures and images for: Acupuncture for prostatectomy incontinence: study protocol for a multicenter single-blind randomized parallel controlled trial
Source: Trials. 2022 Jan 4;23:9. doi: 10.1186/s13063-021-05805-5 (PMC8725553; doi:10.1186/s13063-021-05805-5)

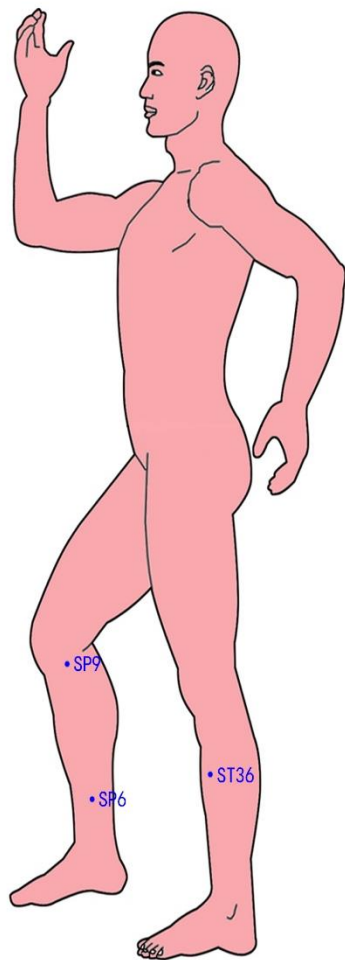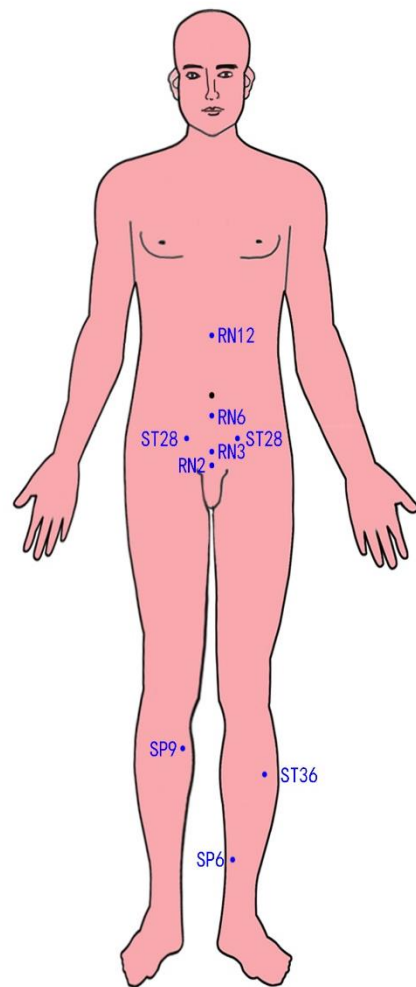

Supplement: Supplementary file 2 — Additional file 2. The acupoint diagram [file 13063_2021_5805_MOESM2_ESM.pdf]
