# Supplementary material for: Acupuncture for prostatectomy incontinence: study protocol for a multicenter single-blind randomized parallel controlled trial
Source: Trials. 2022 Jan 4;23:9. doi: 10.1186/s13063-021-05805-5 (PMC8725553; doi:10.1186/s13063-021-05805-5)
Supplement: Supplementary file 3 — Additional file 3. The ICIQ-UI-SF Scale [file 13063_2021_5805_MOESM3_ESM.pdf]

国际尿失禁咨询委员会尿失禁问卷简表(ICIQ-UI-SF)

许多患者时常漏尿，该表将用于调查尿失禁的发生率和尿失禁对患者的影响程度。仔细回想你近四周来的症状，尽可能回答以下问题。1. 您的出生日期：　　　　2. 性别：

3. 您漏尿的次数? (在一空格内打✓)。	
□ 从来不漏尿。	0
□ 一星期大约漏尿 1 次或经常不到 1 次。	1
□ 一星期漏尿 2 次或 3 次。	2
□ 每天大约漏尿 1 次。	3
□ 一天漏尿数次。	4
□ 一直漏尿。	5
4. 我们想知道您认为自己漏尿的量是多少? 在通常情况下，您的漏尿量是多少(不管您是否使用了防护用品)。(在一空格内打✓)。	
□ 不漏尿。	0
□ 少量漏尿。	2
□ 中等量漏尿。	4
□ 大量漏尿。	6
5. 总体上看，漏尿对您日常生活影响程度如何? 请在 0(表示没有影响)~10(表示有很大影响)之间的某个数字上画圈。<br>0　1　2　3　4　5　6　7　8　9　10<br>没有影响　　　　　　　　　　有很大影响	

ICI-Q-SF 评分(把第 3、4、5 个问题的分数相加):

6. 什么时候发生漏尿?
(请在与您情况相符合的那些□打✓)。
☐ 从不漏尿。
☐ 未能到达厕所就会有尿液漏出。
☐ 在咳嗽或打喷嚏时漏尿。
☐ 在睡着时漏尿。
☐ 在活动或体育运动时漏尿。
☐ 在小便完和穿好衣服时漏尿。
☐ 在没有明显理由的情况下漏尿。
☐ 在所有时间内漏尿。
