# Supplementary material for: Acupuncture for prostatectomy incontinence: study protocol for a multicenter single-blind randomized parallel controlled trial
Source: Trials. 2022 Jan 4;23:9. doi: 10.1186/s13063-021-05805-5 (PMC8725553; doi:10.1186/s13063-021-05805-5)
Supplement: Supplementary file 4 — Additional file 4. 72-hour Voiding Diary [file 13063_2021_5805_MOESM4_ESM.pdf]

72 小时排尿日记卡

以下资料由患者及家属填写：　　　　　　　　　　____年___月___日

日期	时间<br>(点:分)	排尿情况	
		排尿(次)	尿失禁（√）
第一天			
第二天			
第三天			
72 小时尿垫用量：____个			

72h 排尿日记卡汇总

以下资料由医生填写： ___年___月___日

**1. 平均 24h 尿失禁次数：_____ 次**<br>计算方法：72h 内尿失禁次数总和除以 3。
**2. 尿失禁漏尿程度：**<br>□Ⅰ轻度漏尿　□Ⅱ中度漏尿　□Ⅲ重度漏尿<br>Ⅰ（轻度漏尿）：指少量漏尿， 仅漏几滴；尿垫未见成片浸湿（若使用）；<br>Ⅱ（中度漏尿）：指中等量漏尿，漏尿较多，可湿透内裤，但外裤未成片浸湿；可见尿垫成片浸湿（若使用）；<br>Ⅲ（重度漏尿）：指大量漏尿，漏尿多，不仅内裤湿透，同时外裤也成片浸湿；一次漏尿即可将尿垫成片浸湿（若使用）。
**3. 72h 尿垫用量：_____** 个
