# Supplementary material for: Acupuncture for prostatectomy incontinence: study protocol for a multicenter single-blind randomized parallel controlled trial
Source: Trials. 2022 Jan 4;23:9. doi: 10.1186/s13063-021-05805-5 (PMC8725553; doi:10.1186/s13063-021-05805-5)
Supplement: Supplementary file 5 — Additional file 5. The EPIC-26 scale [file 13063_2021_5805_MOESM5_ESM.pdf]

EPIC-26

扩展前列腺癌综合指数

简表

本问卷旨在调查前列腺癌患者的生活水平。请您一定要真实、完整地回答所有问题，以帮助我们获得最精确的数据。

请注意，同病历中的信息一样，本调查中所包含的信息将受到严格保密。

填写日期（请输入调查完成日期）：__________年________月________日

姓名（可不填）：____

出生日期（可不填）：__________年________月________日

勿在本栏作任何标记

1. 在过去4周内，您小便失禁的频率是多久一次？

一天超过一次.........................1

约一天一次 2

一周超过一次.........................3 （请圈选数字） 23/

约一周一次 4

很少或从未出现失禁情况..............5

2. **在过去4周内**，下列哪项最能描述您对小便的控制情况？

无法控制.......................... 1

经常尿滴沥.........................2 （请圈选数字） 26/

偶尔尿滴沥 3

完全控制 4

3. **在过去4周内**，您<u>每天</u>需要用多少卫生垫或成人尿布控制尿滴沥？

无 0
....

每天1片 1

每天2片..................................... 2 （请圈选数字） 27/

每天3片或以上 3

4. **在过去4周内**，下列问题（如果有）对您造成的困扰有多大？

（请在每一行圈选一个数字）

	没有问题	微小问题	小问题	中等问题	大问题	
a. 尿滴沥或漏尿...........	0	1	2	3	4	28/
b. 小便时伴有疼痛或有烧灼感	0	1	2	3	4	29/
c. 小便时出血.............	0	1	2	3	4	30/
d. 小便无力或小便排不净...	0	1	2	3	4	31/
e. 白天要经常小便.........	0	1	2	3	4	33/

5. 总体来说，**在过去4周内**，您的泌尿功能对您造成了多大的困扰？

没有问题 1

微小问题.................................2

小问题...................................3 （请圈选数字） 34/

中等问题 4

大问题 5

版权2002。密西根大学。版权所有。

勿在本栏作任何标记

6. 下列问题（如果有）对您造成的困扰有多大？（请在每一行圈选一个数字）

	没有问题	微小问题	小问题	中等问题	大问题	
a. 排便的程度迫切	0	1	2	3	4	49/
b. 大便次数频繁	0	1	2	3	4	50/
c. 大便失禁	0	1	2	3	4	52/
d. 便 血	0	1	2	3	4	53/
e. 腹部/骨盆/直肠疼痛	0	1	2	3	4	54/

7. 总体来说，**在过去4周内**，您的大便习惯对您造成了多大的困扰？

没有问题 1
微小问题.......................................2
小问题...3 （请圈选数字） 55/
中等问题 4
大问题 5

8. **在过去4周内**，您如何评价下列事项？（请在每一行圈选一个数字）

	很少，几乎没有	差	还行	好	非常好	
a. 您的勃起能力？	1	2	3	4	5	57/
b. 达到性高潮的能力？	1	2	3	4	5	58/

9. **您在过去4周内**的整体勃起质量如何？

根本没有 1
不够坚硬，不适合性行为 2
坚硬度仅限于手淫和前戏 3 （请圈选数字） 59/
坚硬度适合性行为 4

10. 您如何评价您在过**去4周内**的勃起频率？

想要时从未勃起 1
想要时勃起频率不到一半 2
想要时勃起频率约为一半 3 （请圈选数字） 60/
想要时勃起频率多于一半 4
每次想要都能勃起. 5

版权2002。密西根大学。版权所有。

勿在本栏作任何标记

11. **在过去4周内**，您对您整体性功能的评价如何？

很差	1		
差	2		
还可以	3	（请圈选数字）	64/
好	4		
非常好	5		

12. 总体来说，**在过去4周内**，您的性功能或性功能缺乏对您造成了多大的困扰？

没有问题	1		
微小问题	2		
小问题.	3	（请圈选数字）	68/
中等问题	4		
大问题	5		

13. **在过去4周内**，下列问题（如果有）对您造成的困扰有多大？

（请在每一行圈选一个数字）

	没有问题	微小问题	小问题	中等问题	大问题	
a. 潮热	0	1	2	3	4	74/
b. 胸部触痛/肿胀	0	1	2	3	4	75/
c. 心情郁闷	0	1	2	3	4	77/
d. 乏力	0	1	2	3	4	78/
e. 体重发生变化	0	1	2	3	4	79/

版权2002。密西根大学。版权所有。

非常感谢！

版权2002。密西根大学。版权所有。
