# Supplementary material for: Acupuncture for prostatectomy incontinence: study protocol for a multicenter single-blind randomized parallel controlled trial
Source: Trials. 2022 Jan 4;23:9. doi: 10.1186/s13063-021-05805-5 (PMC8725553; doi:10.1186/s13063-021-05805-5)
Supplement: Supplementary file 6 — Additional file 6. The Self-Rating Anxiety scale [file 13063_2021_5805_MOESM6_ESM.pdf]

焦虑自评量表（SAS）

在过去 2 周，请选择下列每个叙述适合您的持续时间。

1= 无或很少有　2= 有时　3= 大部分时间　4= 绝大多数时间

	无或很少有			绝大多数时间
1. 我觉得比平常容易紧张和着急	1	2	3	4
2. 我无缘无故的感到害怕	1	2	3	4
3. 我容易心里烦乱或感到惊恐	1	2	3	4
4. 我觉得我可能将要发疯	1	2	3	4
5. 我觉得一切都很好	1	2	3	4
6. 我手脚发抖打颤	1	2	3	4
7. 我因为头痛、头颈痛和背痛而烦恼	1	2	3	4
8. 我感觉容易衰弱和疲乏	1	2	3	4
9. 我觉得心平气和，而且容易安静坐着	1	2	3	4
10. 我觉得心跳得很快	1	2	3	4
11. 我因为一阵阵头晕而苦恼	1	2	3	4
12. 我有晕倒发作或要晕倒似的	1	2	3	4
13. 我吸气呼气都感到很容易	1	2	3	4
14. 我手脚麻木和刺痛	1	2	3	4
15. 我因为胃痛和消化不良而苦恼	1	2	3	4
16. 我常常要小便	1	2	3	4
17. 我的手常常是潮湿的	1	2	3	4
18. 我脸红发热	1	2	3	4
19. 我容易入睡，并且一夜睡的很好	1	2	3	4
20. 我做恶梦	1	2	3	4

评分说明

20 项中 15 项为负性陈述，1-4 分正向计分；5 项为正性陈述，4-1 分反向计分；20 项总分之和为粗分，粗分×1.25=标准分；标准分 50 分为划界分，50-59 分为轻度焦虑，60-69 分为中度焦虑，70 分以上为重度焦虑。结果仅供临床参考。
